# Exploring nonlinearity in quarter car models with an experimental approach to formulating fractional order form and its dynamic analysis

**DOI:** 10.1038/s41598-024-63139-z

**Published:** 2024-05-27

**Authors:** Tadios Molla, Prakash Duraisamy, Karthikeyan Rajagopal, Anitha Karthikeyan, Salah Boulaaras

**Affiliations:** 1 Production Engineering Department, College of Engineering, Defence University, 1041 Bishoftu, Oromiya Ethiopia; 2 Centre for Nonlinear Systems, Chennai Institute of Technology, Chennai, Tamil Nadu 60069 India; 3 Department of Electronics and Communication Engineering, Vemu Institute of Technology, Chitoor, Andhra Pradesh 517112 India; 4 Department of Mathematics, College of Sciences, Qassim University, 51452 Buraydah, Saudi Arabia

**Keywords:** Nonlinear quarter car model, Stochastic excitation, Adam–Bashforth–Moulton method, Dynamical analysis, Nonlinear equations, Engineering, Mathematics and computing, Physics

## Abstract

This study explores the inherent nonlinearity of quarter car models by employing an experimental and numerical approach. The dynamics of vehicular suspension systems are pivotal for ensuring passenger comfort, vehicle stability, and overall ride quality. In this paper we assessed the impact of various parameters and components on suspension performance, enabled the optimization of ride comfort, stability, and handling characteristics. Firstly, experimental analysis allowed for the investigation of factors that are challenging to model theoretically, such as stiffness nonlinearity and damping characteristics, which may vary under different operating conditions. Time domain and frequency response diagram of the model has been obtained. Secondly, a quarter-car with single degree-of-freedom presented and investigated in fractional order form. Fractional order dynamics emphasize nonlinearities in quarter car models, capturing real-world dynamics effectively. The proposed fractional-order nonlinear quarter car model employed Caputo derivative. For numerical analysis of fractional order system, the Adam–Bashforth–Moulton method is used and the disturbance of road assumed to be stochastic. Results show that the dynamic response of the vehicle can be chaotic. Influence of road roughness amplitude and frequency on vehicle vibration is investigated.

## Introduction

The dynamics of vehicular suspension systems play a pivotal role in ensuring passenger comfort, vehicle stability, and overall ride quality. The road excitations may cause shock vibrations, influencing the safety of driving, the comfort of the driver, and the reliability of the suspension system^1–5^. In recent years, the complex and nonlinear dynamic reactions of automotive suspension systems encountering uneven road conditions have garnered escalating attention as a significant area of interest^6–9^. This paper presents an experimental approach to exploring the nonlinearity inherent in quarter car models.

Traditional models often overlook complex interactions and memory effects present in real-world suspension systems. To fill this void, fractional calculus is a promising technique for modeling intricate systems featuring memory effects^10,11^. In modelling actual scenarios of nonlinear suspension system with complicated physical dynamics, fractional calculus is an effective instrument^12–18^.

Considering the inherent fractional characteristics of real systems, it is unsurprising that utilizing fractional order methodologies to depict system dynamics frequently leads to more precise outcomes when contrasted with conventional integer techniques^19–24^. The investigation of fractional calculus in chaotic systems has become increasingly intriguing and presents a promising method for analyzing a variety of real-world systems^25–27^.

The concepts of chaos, stability, and bifurcation are central to the study of nonlinear dynamical systems, offering key insights into the behavior of complex systems and their responses to varying inputs and parameters. Through bifurcation analysis, researchers identified critical points where transitions between different dynamical regimes occur^28,29^. Investigating the interplay between chaos, stability and bifurcation is crucial for comprehending the dynamics of nonlinear systems, as chaotic behavior often coexists with stable regions within the system’s parameter space.

A detailed analysis of the fractional-order damping and hysteresis in a nonlinear quarter-car suspension system is conducted. Stability at equilibrium points is assessed, Lyapunov exponents are calculated, phase portraits are analyzed, bifurcations are explored, and Lyapunov spectra are scrutinized. This study presents and investigates the dynamic characteristics of the proposed fractional-order nonlinear quarter car model, utilizing Caputo derivatives^30^, and employing the Adam–Bashforth–Moulton (ABM) numericalmethod^31^, recognized for its exceptional convergence and accuracy, as elucidated in Ref.^32^.

The novelty of this study lies in its comprehensive investigation of the nonlinearity in quarter car models through an experimental approach and subsequent numerical dynamic analysis, considering stochastic road profiles as input excitation. While previous research has primarily focused on deterministic road conditions, this study uniquely addresses the influence of stochasticity on the dynamic behavior of vehicle suspension systems. By incorporating fractional order modeling and dynamic analysis techniques, the study offers a novel perspective on how nonlinearity interacts with stochastic road disturbances, providing valuable insights into the performance of suspension systems under real-world driving conditions. This approach not only advances the understanding of vehicle dynamics but also informs the development of more robust and adaptive suspension designs capable of effectively handling the uncertainties inherent in road environments.

The structure of this paper is outlined as follows. “Experimental setup and response analysis” section presents the experimental setup and response Analysis. This is followed by mathematical modeling of nonlinear quarter car suspension (“Mathematical modeling of nonlinear quarter car suspension” section) and dynamical analysis (“Dynamic analysis” section). Formulation of quarter car model with fractional model is shown in “Formulation of quarter car model with fractional order” section. “Conclusion” section concludes the paper.

## Experimental setup and response analysis

The objectives of experimental analysis for the quarter car model are multifaceted. Firstly, it provides an avenue for assessing the impact of various parameters and components on suspension performance, enabling the optimization of ride comfort, stability, and handling characteristics. Secondly, experimental analysis allows for the investigation of factors that are challenging to model theoretically, such as friction, wear, and damping characteristics, which may vary under different operating conditions.

This research endeavor embarks on an experimental analysis of the quarter car model, with the primary goal of gaining a deeper understanding of the dynamic behavior of vehicle suspensions. By conducting physical experiments on quarter car setups equipped with sensors to measure displacement, velocity, and acceleration responses, we aim to replicate real-world driving scenarios and collect empirical data. These experiments will encompass a stochastic excitation condition to assess how the suspension system responds to diverse challenges, from smooth highways to rough terrain.

**Figure 1:**
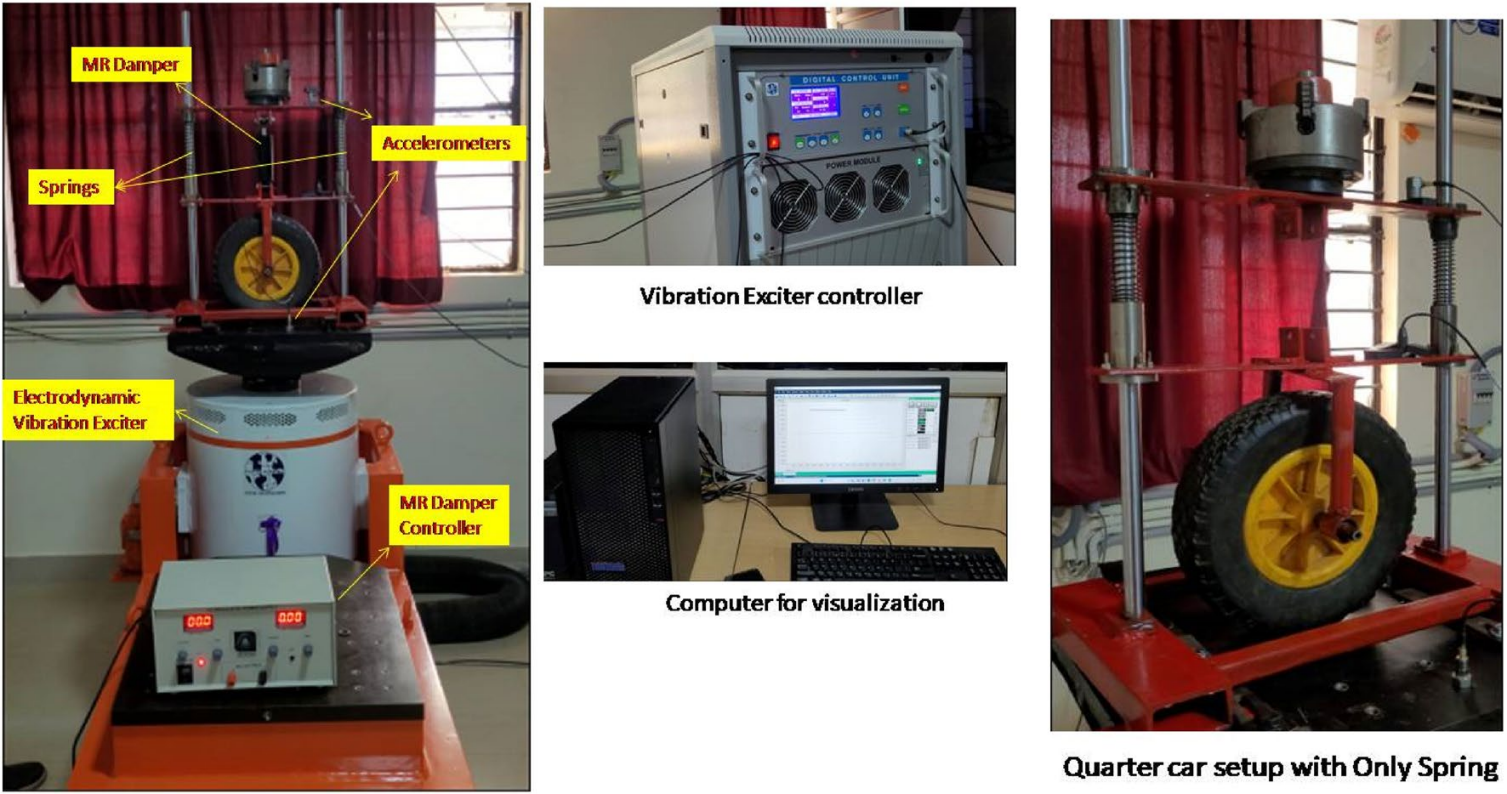
Experimental setup with electrodynamic vibration exciter, accelerometers, controllers and other related components.

The following experimental setup is used to conduct the testing, it consists of (i) Vibration Exciter, (ii) Quarter car model, (iii) Springs with different spring constants, (iv) Magneto rheological (MR) damper, (v) Vibration sensors, (vi) Data Acquisition system, (vii) Software for visualization as shown in Fig. 1 below.

### Response of the system for stiffness nonlinearity

**Figure 2:**
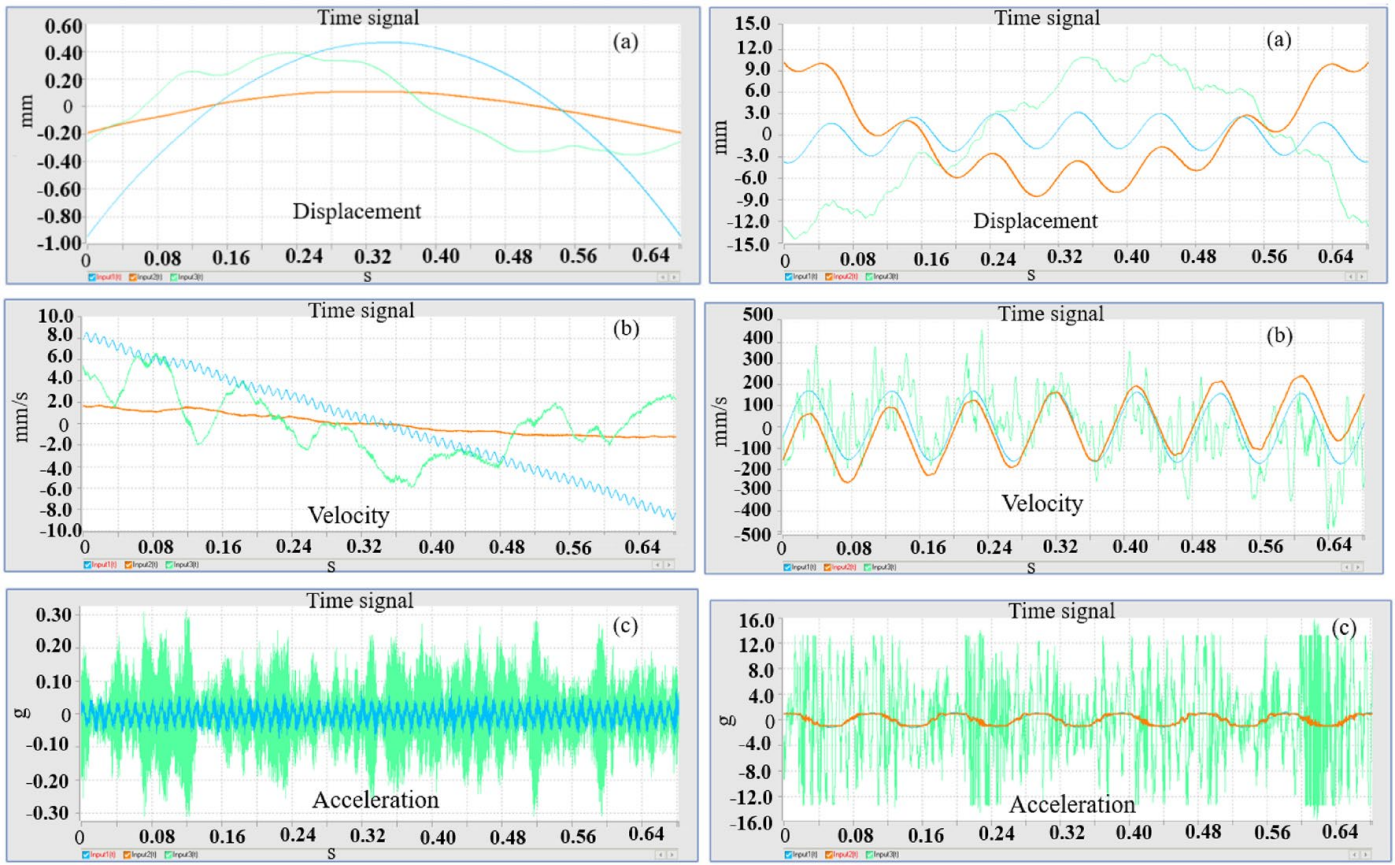
Displacement, velocity and acceleration plots for different stiffness scenario.

In order to understand the influence of parameters affecting the performance of the system, we used two types of springs; the following experimental results are obtained. Figure 2 indicated that the response of the system as displacement, velocity and acceleration.

The results confirm that the response of the system entirely different for different stiffness scenario, using a single spring constant such as in linear model may not valid for real-time implementation. The nonlinear model of the spring should be considered for simulation; hence we used higher order nonlinearity for stiffness for numerical simulations.

**Figure 3:**
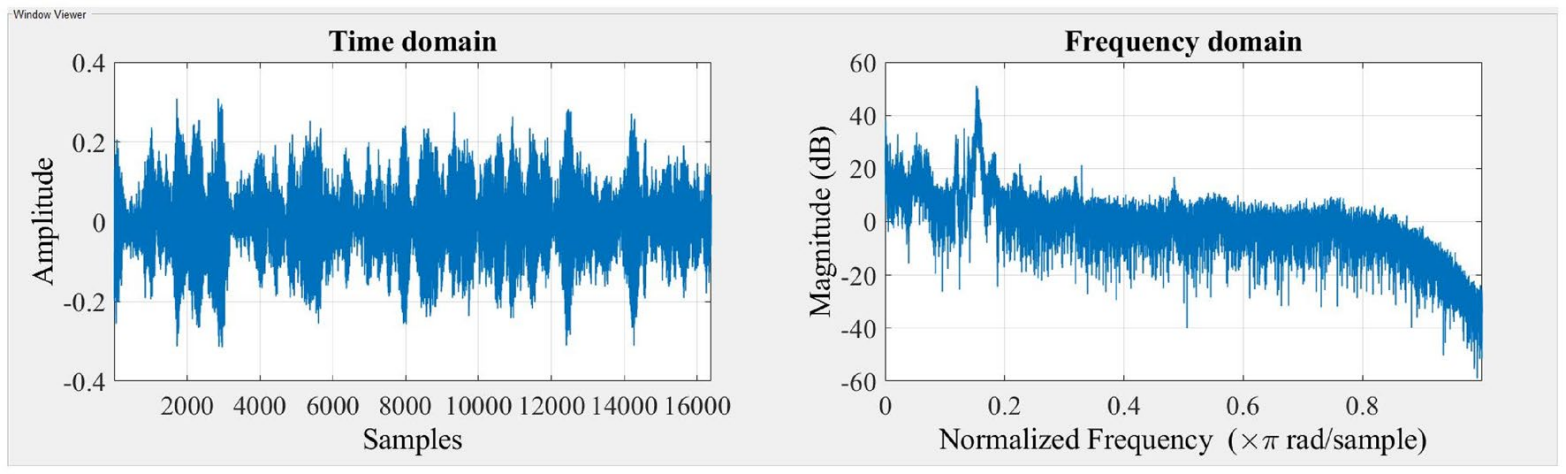
Response of the system for stiffness scenario 1.

**Figure 4:**
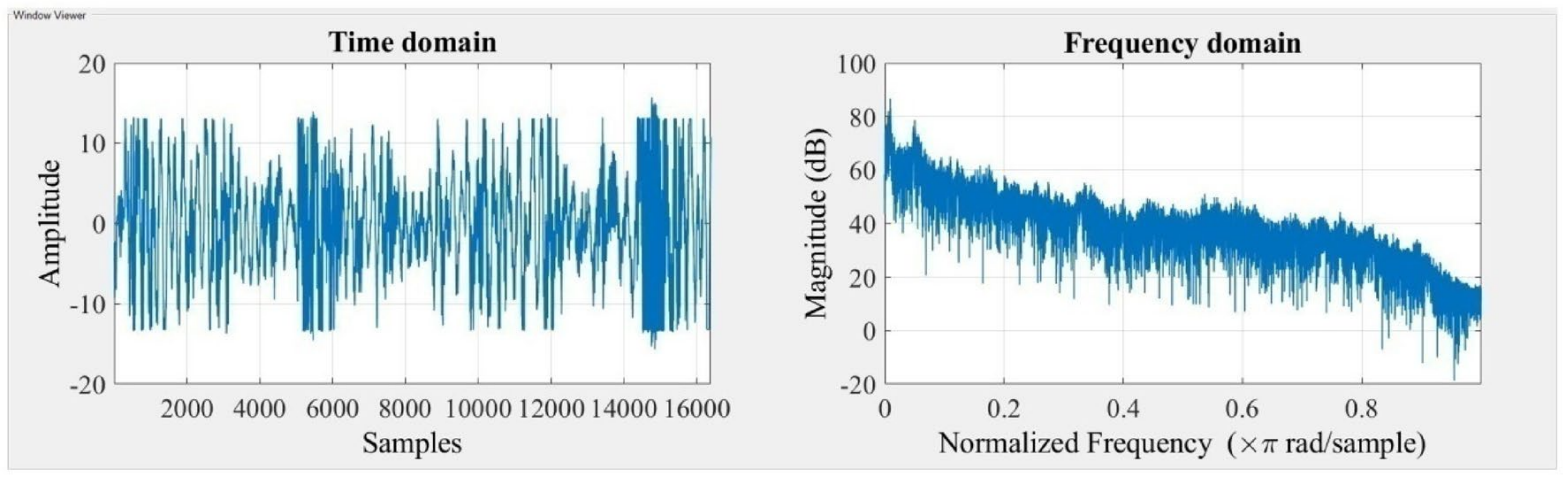
Response of the system for stiffness scenario 2.

The accelerometer records vibration signals, which are then gathered and visually depicted in the Figs. 3 and 4 indicated below. The time domain and frequency domain plots provide a comprehensive representation of the system’s response under two different stiffness scenarios, namely Scenario 1 and Scenario 2.

### Damper nonlinearity

**Figure 5:**
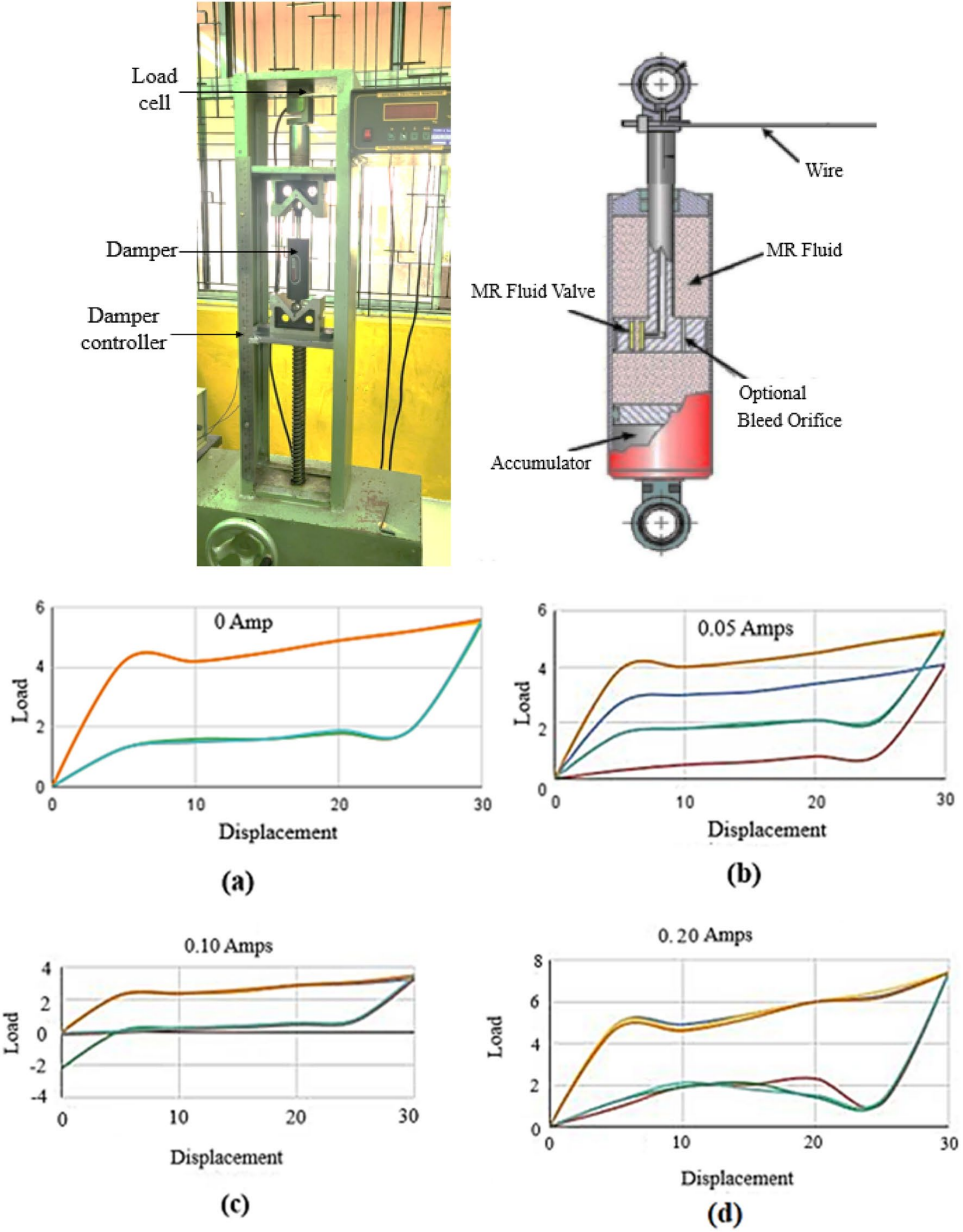
MR Damper characteristic study setup and load–displacement curves.

The magnetorheological Damper (MR) characteristic study setup and load–displacement curves is shown in Fig. 5 below. The presence of nonlinearity in damper is confirmed from the following experiment. The load–displacement curve is plotted for different current scenario of MR damper is presented.

**Figure 6:**
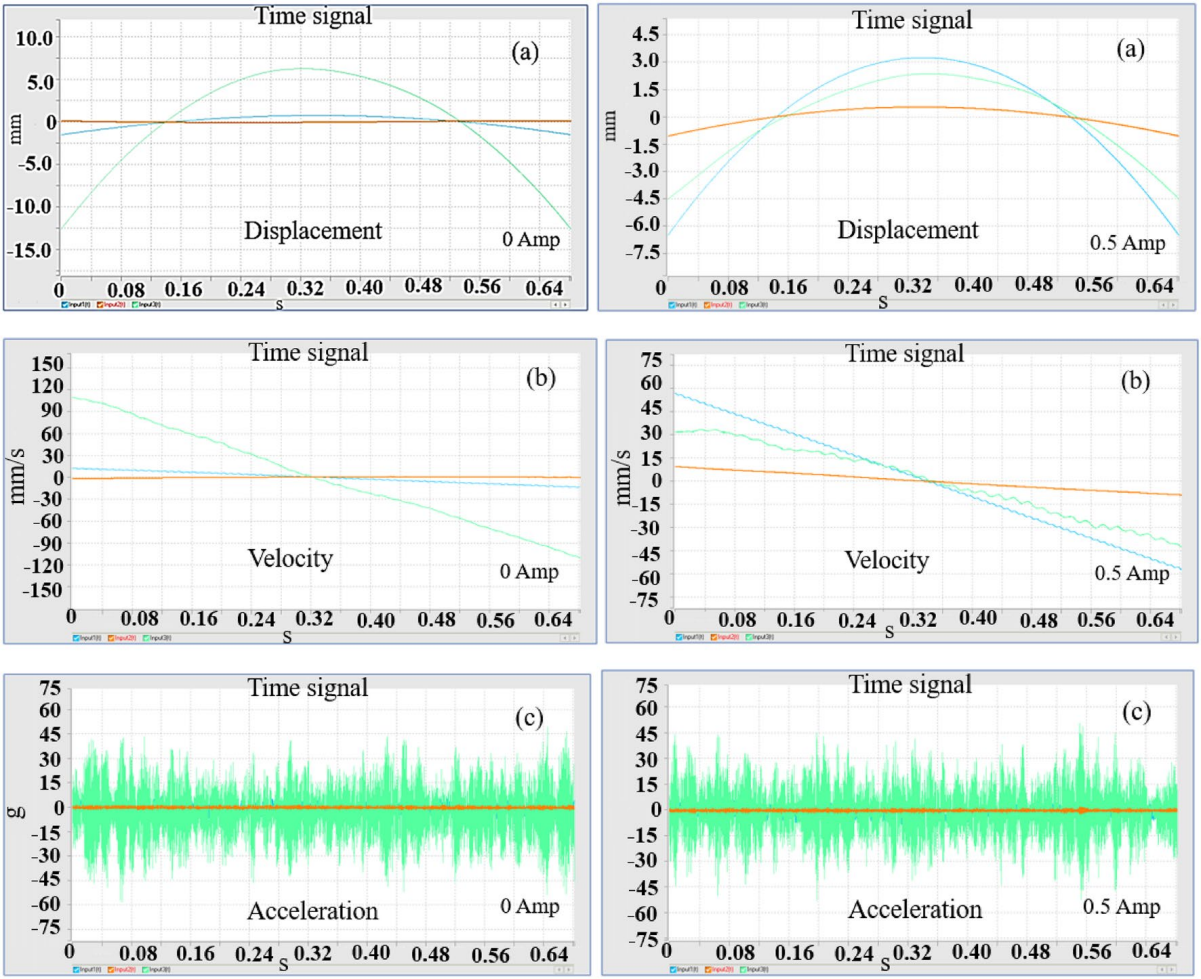
Displacement, velocity, acceleration plots obtained from the experimental setup.

To investigate the integration of MR dampers into the quarter car model and its influence on the dynamic response of the suspension system, we incorporated the MR damper in the Quarter car setup with a focus on measuring essential parameters such as displacement, velocity, and acceleration as shown in Fig. 6 below.

**Figure 7:**
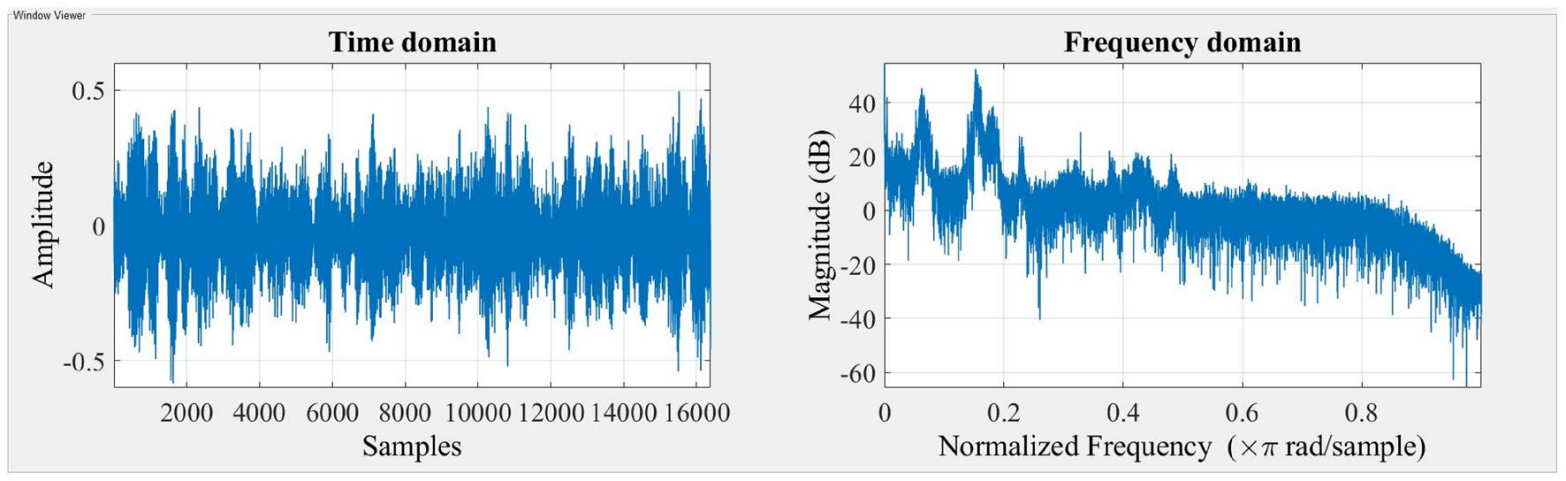
Response of the system with Damper (0 A).

**Figure 8:**
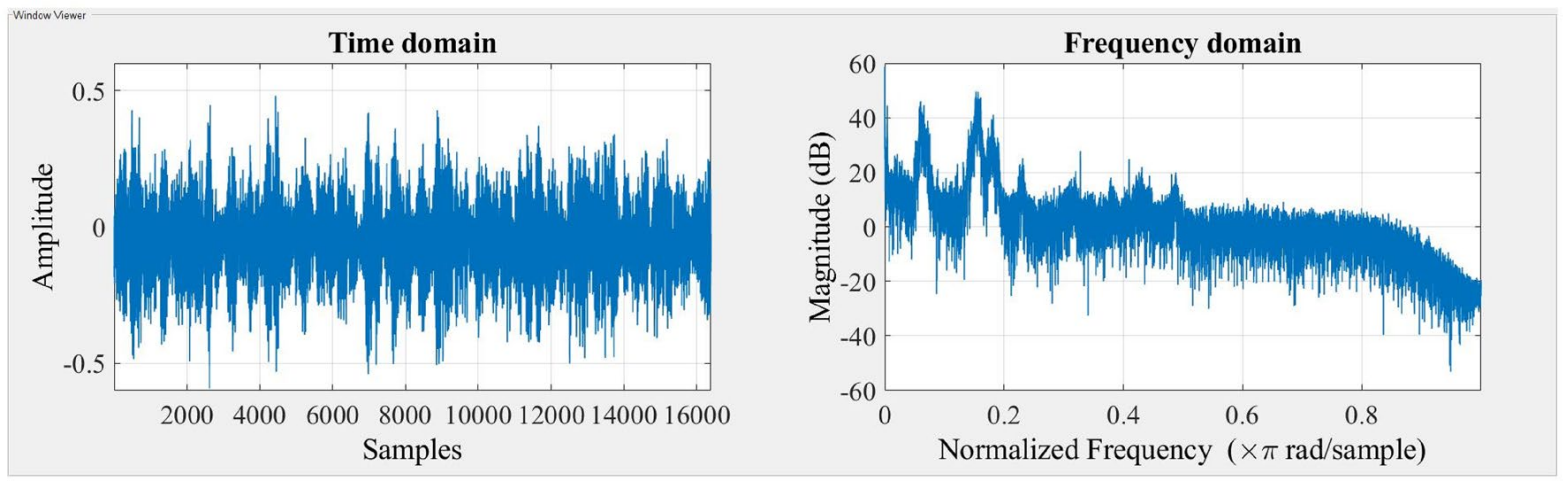
Response of the system with Damper (0.2 A).

**Figure 9:**
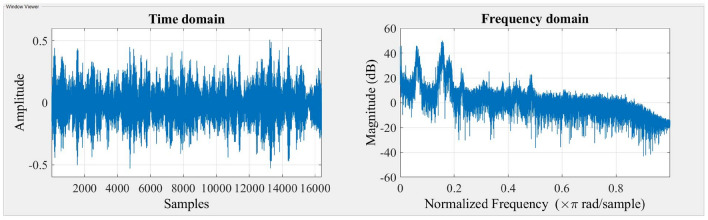
Response of the system with Damper (0.5 A).

The time domain response provides insights into the system’s transient behavior and damping effects over time, while the frequency domain response analyzes the system’s behavior in terms of frequency components and resonances. Together, these responses help engineers design and fine-tune suspension systems equipped with MR dampers to optimize ride comfort, handling, and overall vehicle performance under diverse driving conditions. The time domain response and frequency domain response are presented in Figs. 7, 8 and 9.

## Mathematical modeling of nonlinear quarter *car* suspension

**Figure 10:**
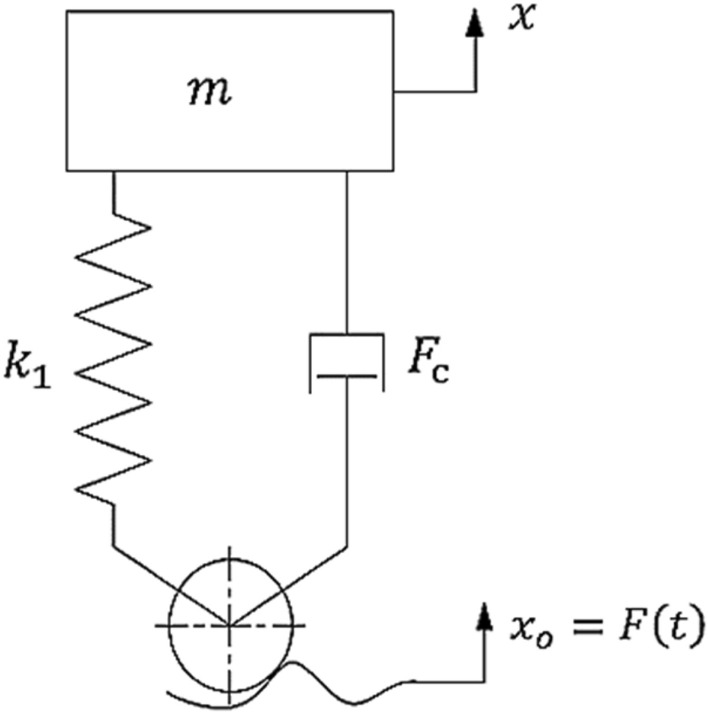
Schematic diagram of SDOF quarter car model.

Figure 10 presents a single degree of freedom (SDOF) quarter-car model with hysteretic nonlinear damping. The integer-order differential equation of motion is established first. Then, based on Caputo’s fractional derivative, the fractional order quarter car model is presented.

The following is established for the integer order of the suspension system dynamical equation^33^:

1 $$m\ddot{x} + k_{1} \left( {x - x_{o} } \right) + F_{c} = 0,$$

where $m$ is mass of the body,$\ddot{x}$ is the vertical acceleration of the mass, $k_{1} = 16,000\;{\text{N}}\;{\text{m}}^{ - 1}$ is the suspension stiffness coefficient,$x_{o}$ the road excitation, *x* the body’s vertical displacement and $F_{c}$ is hysteretic nonlinear damping and stiffness force, which is dependent on the relative displacement and velocity given by:

2 $$F_{c} = k_{2} \left( {x - x_{o} } \right)^{3} + c_{1} \left( {\dot{x} - \dot{x}_{o} } \right) + c_{2} \left( {\dot{x} - \dot{x}_{o} } \right)^{3} ,$$

where $m = 240\;{\text{kg}}$ and $k_{2} = - 30,000\;{\text{N}}\;{\text{m}}^{ - 3}$, $c_{1} = 250\;{\text{N}}\;{\text{s}}\;{\text{m}}^{ - 3}$ and $c_{2} = - 25\;{\text{N}}\;{\text{s}}^{ - 3} \;{\text{m}}^{ - 3}$ are hysteretic nonlinear damping force with constants^33^.

Considering Eqs. (1), (2) can be rewritten as

3 $$m\ddot{x} + k_{1} \left( {x - x_{o} } \right) + k_{2} \left( {x - x_{o} } \right)^{3} + c_{1} \left( {\dot{x} - \dot{x}_{o} } \right) + c_{2} \left( {\dot{x} - \dot{x}_{o} } \right)^{3} = 0.$$

Taking into account the relative vertical displacement $y = x - x_{o}$, and the road profile $x_{o} = F\left( t \right)$, where $F\left( t \right)$ is a three-dimensional chaotic system^34^ as stochastic excitation.

We are able to reduce Eq. (3) to a simpler form, which is:

4 $$\ddot{y} + \sigma y + \beta y^{3} + \gamma \dot{y} + \delta \dot{y}^{3} = F\left( t \right),$$

where $$\sigma = \frac{{k_{1} }}{m},\,\beta = \frac{{k_{2} }}{m},\gamma = \,\frac{{c_{1} }}{m}\,and\,\delta = \frac{{c_{2} }}{m}$$. The states are chosen such that $$y_{1} = y,\,$$$$y_{2} = \dot{y}.$$ Thus, the system’s state space representation can be expressed as follows:

5 $$\begin{array}{*{20}l} {\dot{y}_{1} = y_{2} ,} \hfill \\ {\dot{y}_{2} = - \sigma y_{1} - \beta y_{1}^{3} - \gamma y_{2} - \delta y_{2}^{3} + F(t).} \hfill \\ \end{array}$$

## Dynamic analysis

### Equilibrium points

The dynamic analysis starts with finding the equilibrium points stability. Since it is a nonlinear equation, it may have more than one equilibrium point. Let us found it by setting LHS of the equations equal to zero.

6 $$\begin{gathered} 0 = y_{2} , \hfill \\ 0 = - \sigma y_{1} - \beta y_{1}^{3} - \gamma y_{2} - \delta y_{2}^{3} . \hfill \\ \end{gathered}$$

The system exhibits a finite number of equilibrium points, which can be determined as follows: $E_{1} = \left[ { - 0.7303,\,0} \right]$, $E_{2} = \left[ {0.7303,\,0} \right]$ and $E_{3} = \left[ {0,\,0} \right]$.

### Stability analysis

The Jacobian matrix for the given quarter car model at its equilibrium positions can be computed as follows:

7 $$J = \left[ {\begin{array}{*{20}c} 0 & 1 \\ { - 3\beta y_{1}^{2} - \sigma } & {3\delta y_{2}^{2} - \gamma } \\ \end{array} } \right].$$

The characteristics equation is determined through the expression $\det \left[ {\lambda I - J} \right] = 0.$ Solving this characteristic equation for each equilibrium point enables us to obtain the eigenvalues of the system, for the integer-order model of the first degree of freedom suspension system (5), when the commensurate order of the system $\alpha = 1$, the characteristic equation of the system is derivedas $\lambda^{2} + a_{1} \lambda \pm a_{3} ,$ and at equilibrium E_1_ and E_2_, the characteristic equation is $\lambda^{2} + 1.0417\lambda - 133.335$, and the corresponding eigenvalues are given in Table 1, and $\lambda_{1}$ is the saddle node. Similarly, at equilibrium E_3_, the characteristic equation is $\lambda^{2} + 1.0417\lambda + 66.667$, and the corresponding eigenvalues are given in Table 1, where $\lambda_{1,2}$ is the stable spiral. As per the Routh-Hurwitz criterion, all the principal minors need to be positive, but at equilibrium, E_1_ and E_2_ fail this condition. At this equilibrium point, the system (5) is unstable and shows chaotic oscillations. In a similar way, all the principal minors at equilibrium E_3_ are positive; at this equilibrium point, the system (5) is stable.

**Table 1:** The equilibrium points and Eigen values of the System.

S. no.	Equilibrium points	Eigen values	Stability
1	$E_{1} = \left[ { - 0.7303,\,0} \right]$	$\lambda_{1} = 11.0380,\,\lambda_{2} = - 12.0797$	Saddle node
2	$E_{2} = \left[ {0.7303,\,0} \right]$	$\lambda_{1} = 11.0380,\,\lambda_{2} = - 12.0797$	Saddle node
3	$E_{3} = \left[ {0,\,0} \right]$	$\lambda_{1,2} = - 0.5209 \pm 8.1483i$	Stable spiral

As shown in the Table 1, the Eigen values $\lambda_{1}$ of the equilibrium points $E_{1}$ and $E_{2}$ are saddle unstable points and the Eigen values $\lambda_{1,2}$ of the equilibrium points $E_{3}$ are stable which satisfy the stability condition for chaotic behavior.

### Response of the system for stochastic excitation

Considering $F\left( t \right)$ a three-dimensional chaotic system^34^ as stochastic excitation:

8 $$\begin{array}{*{20}l} \dot{u} = au - dvw, \hfill \\ \dot{v} = - bv + uw, \hfill \\ \dot{w} = - cw + uvw + k. \hfill \\ \end{array}$$

Considering the state value of $w$ is fetched as stochastic excitation in the system (5), then it can be written as:

9 $$\begin{array}{*{20}l} \dot{y}_{1} = y_{2} , \hfill \\ \dot{y}_{2} = - \sigma y_{1} - \beta y_{1}^{3} - \gamma y_{2} - \delta y_{2}^{3} + ew, \hfill \\ \end{array}$$

where $e$ the tuning parameter and *w* is the stochastic noise.

**Figure 11:**
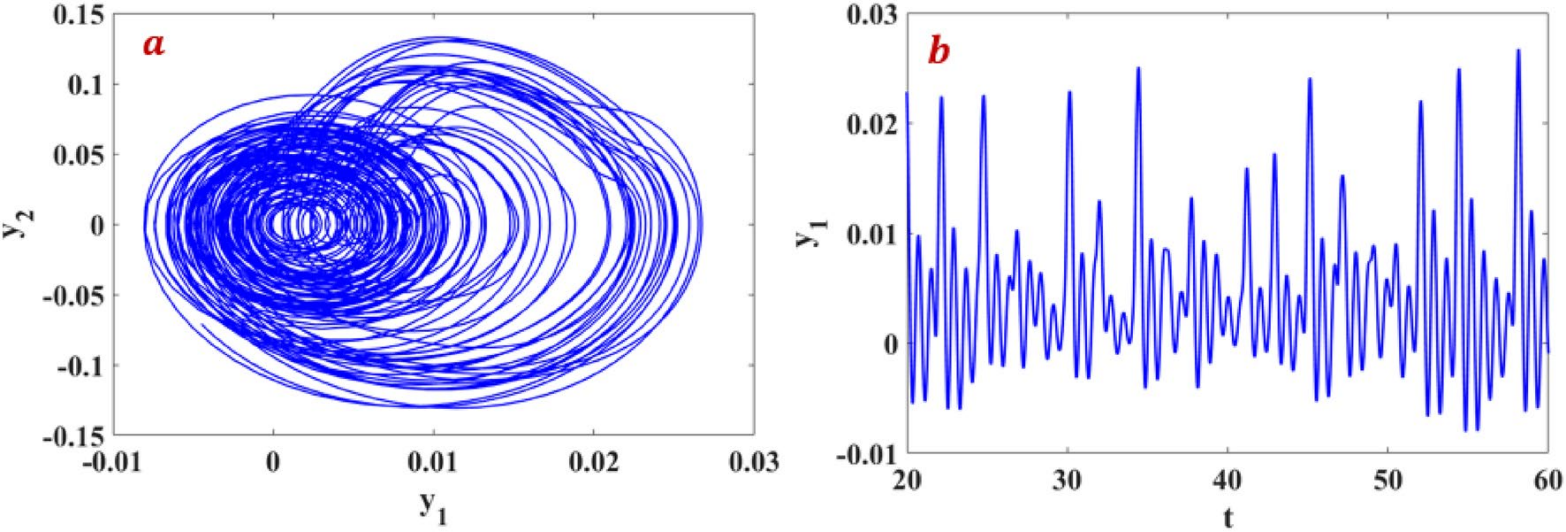
Phase Portrait and time domain response for the system (5) under integer order. (**a**) Phase on x–y plane and (**b**) time domain response.

The phase portraits and time series of system (9) is shown in Fig. 11a,b. For parameter values $\sigma = 66.6667$, $\beta = - 125$, $\gamma = 1.0417$, $\delta = - 0.1042$, $c = 4$, $d = 1$, $a = 2$, $b = 7$, $k = 4$, $e = 0.06$, and the initial conditions $\left[ {0,\,0.1,\,0.1,\,0.1,\,0.1} \right]$ the system exhibits chaotic behaviour.

## Formulation of quarter *car* model with fractional order

Fractional calculus has a long history, stretching back over three hundred years. It is a non-integer order fundamental operator generalization of integration and differentiation, denoted by ${}_{a}D_{t}^{\alpha }$, where $a$ and $t$ are the operator’s limits^35^.

10 $${}_{a}D_{t}^{\alpha } = \left\{ \begin{gathered} \frac{{d^{\alpha } }}{{dt^{\alpha } }}\,\,\,\,\,\,\,\,\,\,\,\,\,\,\,\,\,\alpha > 0 \hfill \\ 1\,\,\,\,\,\,\,\,\,\,\,\,\,\,\,\,\,\,\,\,\,\,\alpha = 0 \hfill \\ \int_{a}^{t} {\left( {d\tau } \right)^{ - \alpha } \,\,\,\,\,\,\alpha < 0} \hfill \\ \end{gathered} \right..$$

There exist three commonly used definitions for the fractional order differential operator, viz. Grunwald–Letnikov, Riemann–Liouville and Caputo^36^. Caputo type fractional calculus is used in this paper which is defined as

11 $${}_{a}D_{t}^{\alpha } g\left( t \right) = \frac{1}{{\Gamma \left( {p - \alpha } \right)}}\int_{a}^{t} {\frac{{f^{\left( p \right)} \left( \tau \right)}}{{\left( {t - \tau } \right)^{\alpha - p + 1} }}d\tau ,\,\,\,\,\,\,for\,\,p - 1 < \alpha < p} ,$$

where $\alpha$ is the order.

By incorporating these fractional order approximations into the integer order model (Eq. 9), we obtain the fractional order nonlinear quarter car model as described in Eq. (12).

12 $$\begin{array}{*{20}l} D_{t}^{\alpha } y_{1} = y_{2} , \hfill \\ D_{t}^{\alpha } y_{2} = - \sigma y_{1} - \beta y_{1}^{3} - \gamma y_{2} - \delta y_{2}^{3} + ew, \hfill \\ \end{array}$$

where α is the fractional order σ β, γ, δ, a, b, c, d, k, are parameters and e the tuning parameter and $w$ is the stochastic noise. The stability analysis of fractional-order systems based on eigenvalues is an important approach, similar to the way eigenvalues are used in analyzing stability for integer-order systems. The fraction order for every eigenvalue is obtained using Eq. (13). The greatest value among the obtained fractional order is selected as the fraction order of the system. In a dynamic system with the size of n, the eigenvalues, $\left( {\lambda_{1,} \lambda_{2} ,....\lambda_{n} } \right)$, are obtained using Jacobian matrices created for each dimension. Then, every single eigenvalue is used to determine the fraction order, $\alpha$, using (13), where $\alpha$ is expressed as the determined fraction order.

13 $$\left| {\arg \left( {\lambda_{i} } \right)} \right| \succ \frac{\alpha \pi }{2},\,\,\,\,\,\,\alpha = \max \left( {\alpha_{1} ,\alpha_{2} ,....\alpha_{n} } \right),\,\,\,\forall \left( {i = 1,2...,n} \right).$$

As the Table 1 illustrates, the eigenvalues for the Jacobian matrix at equilibrium point $E_{3}$ of a given system (12) are $\lambda_{1} = - 0.5209 + 8.1483i$, $\lambda_{2} = - 0.5209 - 8.1483i$. In such a case, a fractional order needs to be calculated for the corresponding eigenvalue: $\arg \left( {\lambda_{1} } \right) = - 1.5069$ and $\arg \left( {\lambda_{2} } \right) = 1.5069.$ Then, fractional order using the expression $\max \left( {\alpha_{1} ,\alpha_{2} ,....\alpha_{n} } \right)$ provides $\alpha = \alpha_{1} = \alpha_{2} = 0.98$. In the case $\alpha_{1} ,$ and $\alpha_{2}$ are greater than 0.95.

### Numerical algorithm for fractional-order nonlinear quarter car model

Various methodologies are used for the numerical analysis of the fractional order systems^36^. We are considering the Adam–Bashforth–Moulton (ABM) approach.

Consider *α* as the order a fractional order dynamical system

14 $$D^{\alpha } x = f\left( {t,x} \right),\,\,\,0 \le t \le T,$$

and $y^{k} \left( 0 \right) = y_{o}^{k}$ for $k \in \left[ {0,n - 1} \right]\,$, *T* can be the finite time.

Equation (12) approaches the expression for the Volterra integral given in Ref.^37^;

15 $$x\left( t \right) = \sum\limits_{k = 0}^{n - 1} {z_{o}^{k} } \frac{{t^{k} }}{k!} + \frac{1}{\Gamma \left( \alpha \right)}\int\limits_{0}^{t} {\frac{{f\left( {\tau_{1} ,x} \right)}}{{\left( {t - x} \right)}}} d\tau_{1} ,$$

where $$h = \frac{T}{N},\,t_{n} = nh:\,\,h \in \left[ {0,N} \right]$$ and $$\tau_{1} = \frac{\tau }{h}$$.

We can define the discrete form of Eq. (14) as,

16 $$x\left( {n + 1} \right) = \sum\limits_{0}^{n - 1} {x_{o}^{k} } \frac{{t^{k} }}{k!} + \frac{{h^{\alpha } }}{{\tau \left( {\alpha + z} \right)}}f\left( {t_{n + 1} ,x_{n}^{\prime} \left( {n + 1} \right) + \frac{{h^{\alpha } }}{{\Gamma \left( {\alpha + z} \right)}}\sum {a_{j,n + 1} } f\left( {t_{j} ,x_{n} \left( j \right)} \right)} \right),$$

where

17 $$a_{j,n + 1} = \left\{ {\begin{array}{*{20}l} {n^{\alpha + 1} - \left( {\left( {n - \alpha } \right)n + 1} \right)^{\alpha + 1} } \hfill & {j = 0} \hfill \\ {\left( {n - j + 2} \right)^{\alpha + 1} + \left( {n - j} \right)^{\alpha + 1} - 2\left( {n - j + 1} \right)^{\alpha + 1} } \hfill & {1 \le j < n} \hfill \\ 1 \hfill & {j = n + 1} \hfill \\ \end{array} } \right.,$$

18 $$x_{n}^{\prime} \left( {n + 1} \right) = \sum\limits_{k = 0}^{n - 1} {x_{o}^{k} } \frac{{t^{k} }}{k!} + \frac{1}{\Gamma \left( \alpha \right)}\sum\limits_{j = 0}^{n} {b_{j,n + 1} f\left( {t_{j} ,z_{n} \left( j \right)} \right)} ,$$

19 $$b_{j,n + 1} = \frac{{h^{\alpha } }}{\alpha }\left( {\left( {n - j + 1} \right)^{\alpha } - \left( {n - j} \right)^{\alpha } } \right).$$

Using the definitions of (15) and (16), the fractional order nonlinear quarter car model can be defined as,

20 $$y_{1} \left( {n + 1} \right) = y_{1} \left( 0 \right) + \frac{{h^{{\alpha_{{y_{1} }} }} }}{{\Gamma \left( {\alpha_{{y_{1} }} + 2} \right)}}\left( {\left( {y^{\prime}_{2} \left( {n + 1} \right)} \right) + \sum\limits_{j = 0}^{n} {x_{1,j,n + 1} } \left( {y^{\prime}_{2} \left( j \right)} \right)} \right),$$

21 $$y_{2} \left( {n + 1} \right) = y_{2} \left( 0 \right) + \frac{{h^{{\alpha_{{y_{2} }} }} }}{{\Gamma \left( {\alpha_{{y_{2} }} + 2} \right)}}\left( \begin{gathered} - \sigma \left( {y^{\prime}_{1} \left( {n + 1} \right)} \right) - \beta \left( {y^{\prime}_{1} \left( {n + 1} \right)} \right)^{3} - \gamma \left( {y^{\prime}_{2} \left( {n + 1} \right)} \right) \hfill \\ - \delta \left( {y^{\prime}_{2} \left( {n + 1} \right)} \right)^{3} + e\left( {y^{\prime}_{5} \left( {n + 1} \right)} \right) \hfill \\ + \sum\limits_{j = 0}^{n} {x_{2,j,n + 1} } \left( \begin{gathered} \sigma \left( {y^{\prime}_{1} \left( j \right)} \right) - \beta \left( {y_{1}^{\prime} \left( j \right)} \right)^{3} - \gamma \left( {y_{2} \left( j \right)} \right) \hfill \\ - \delta \left( {y_{2}^{\prime} \left( j \right)} \right)^{3} + e\left( {y^{\prime}_{5} \left( j \right)} \right) \hfill \\ \end{gathered} \right) \hfill \\ \end{gathered} \right),$$

22 $$u\left( {n + 1} \right) = u\left( 0 \right) + \frac{{h^{{\alpha_{u} }} }}{{\Gamma \left( {\alpha_{u} + 2} \right)}}\left( \begin{gathered} a\left( {u^{\prime}\left( {n + 1} \right)} \right) - d\left( {v^{\prime}\left( {n + 1} \right)} \right)w^{\prime}\left( {n + 1} \right) \hfill \\ + \sum\limits_{j = 0}^{n} {x_{3,j,n + 1} } \left( {a\left( {u^{\prime}\left( j \right)} \right) - d\left( {v^{\prime}\left( j \right)} \right)w^{\prime}\left( j \right)} \right) \hfill \\ \end{gathered} \right),$$

23 $$v\left( {n + 1} \right) = v\left( 0 \right) + \frac{{h^{{\alpha_{v} }} }}{{\Gamma \left( {\alpha_{v} + 2} \right)}}\left( \begin{gathered} - b\left( {v^{\prime}\left( {n + 1} \right)} \right) + u^{\prime}\left( {n + 1} \right)w^{\prime}\left( {n + 1} \right) \hfill \\ + \sum\limits_{j = 0}^{n} {x_{4,j,n + 1} } \left( { - b\left( {v^{\prime}\left( j \right)} \right) + u^{\prime}\left( j \right)w^{\prime}\left( j \right)} \right) \hfill \\ \end{gathered} \right),$$

24 $$w\left( {n + 1} \right) = w\left( 0 \right) + \frac{{h^{{\alpha_{w} }} }}{{\Gamma \left( {\alpha_{w} + 2} \right)}}\left( \begin{gathered} c\left( {w^{\prime}\left( {n + 1} \right)} \right) + u^{\prime}\left( {n + 1} \right)v^{\prime}\left( {n + 1} \right)w^{\prime}\left( {n + 1} \right) + k \hfill \\ + \sum\limits_{j = 0}^{p} {x_{5,j,n + 1} } \left( {c\left( {w^{\prime}\left( j \right)} \right) + u^{\prime}(j)v^{\prime}(j)w^{\prime}\left( j \right) + k} \right) \hfill \\ \end{gathered} \right),$$

25 $$y_{1}^{\prime} \left( {n + 1} \right) = y_{1} \left( 0 \right) + \frac{1}{{\Gamma \left( {\alpha_{{y_{1} }} + 2} \right)}}\left( {\sum\limits_{j = 0}^{n} {\theta_{1,j,n + 1} } \left( {y_{2} \left( j \right)} \right)} \right),$$

26 $$y_{2}^{\prime} \left( {n + 1} \right) = y_{2} \left( 0 \right) + \frac{1}{{\Gamma \left( {\alpha_{{y_{2} }} + 2} \right)}}\left( {\sum\limits_{j = 0}^{n} {\theta_{2,j,n + 1} } \left( \begin{gathered} - \alpha \left( {y_{1} \left( j \right)} \right) - \beta \left( {y_{1} \left( j \right)^{3} } \right) - \gamma \left( {y_{2} \left( j \right)} \right) \hfill \\ - \delta \left( {y_{2} \left( j \right)^{3} } \right) + \left( {e\left( {y_{5} \left( j \right)} \right)} \right) \hfill \\ \end{gathered} \right)} \right),$$

27 $$u^{\prime}\left( {n + 1} \right) = u\left( 0 \right) + \frac{1}{{\Gamma \left( {\alpha_{u} + 2} \right)}}\left( {\sum\limits_{j = 0}^{n} {\theta_{3,j,n + 1} } \left( {a\left( {u\left( j \right)} \right) - d\left( {v\left( j \right)} \right)\left( {w\left( j \right)} \right)} \right)} \right),$$

28 $$v^{\prime}\left( {n + 1} \right) = v\left( 0 \right) + \frac{1}{{\Gamma \left( {\alpha_{v} + 2} \right)}}\left( {\sum\limits_{j = 0}^{n} {\theta_{4,j,n + 1} } \left( { - b\left( {v\left( j \right)} \right) + u\left( j \right)w\left( j \right)} \right)} \right),$$

29 $$w^{\prime}\left( {n + 1} \right) = w\left( 0 \right) + \frac{1}{{\Gamma \left( {\alpha_{w} + 2} \right)}}\left( {\sum\limits_{j = 0}^{n} {\theta_{5,j,n + 1} } \left( {c\left( {w\left( j \right)} \right) + u\left( j \right)v\left( j \right)w\left( j \right)} \right)} \right),$$

30 $$x_{i,j,n + 1} = \left\{ {\begin{array}{*{20}l} {n^{\alpha + 1} - \left( {n - \alpha } \right)\left( {n + 1} \right)^{\alpha + 1} } \hfill & {j = 0} \hfill \\ {\left( {n - j + 2} \right)^{\alpha + 1} + \left( {n - j} \right)^{\alpha + 1} - 2\left( {n - j + 1} \right)^{\alpha + 1} } \hfill & {1 \le j < n} \hfill \\ 1 \hfill & {j = n + 1} \hfill \\ \end{array} } \right.,$$

31 $$\theta_{i,j,n + 1} = \frac{{h^{\alpha } }}{\alpha }\left( {\left( {n - j + 1} \right)^{\alpha } - \left( {n - j} \right)^{\alpha } } \right),\,\,\,\,\,0 \le j \le n\,\,for\,i = 1,2,3,4,5.$$

### Lyapunov exponents

Using the Wolf’s algorithm^34^ and modified Wolf’s algorithms^38^, we calculate the finite time Lyapunov exponents (LEs) of the system (12). The calculations carried out with the initial conditions as $$\left[ {0,0.1,0.1,0.1,0.1} \right]$$ and finite time duration for LEs computation is set at 20,000 s. The LEs of the system for fractional order α = 0.998 is calculated as $$L_{1} = - 0.5411,$$$$L_{2} = - 0.5413,$$$$L_{3} = 0.7675,$$$$L_{4} = - 0.0594\,and$$ $$L_{5} = - 7.1908.$$ Since the system has one positive Lyapunov exponent, it is categorized as chaotic system and $$L_{1} + L_{2} + L_{3} + L_{4} + L_{5} = - 8.3326 < 0$$, this shows that the system is dissipative.

### Phase portrait

**Figure 12:**
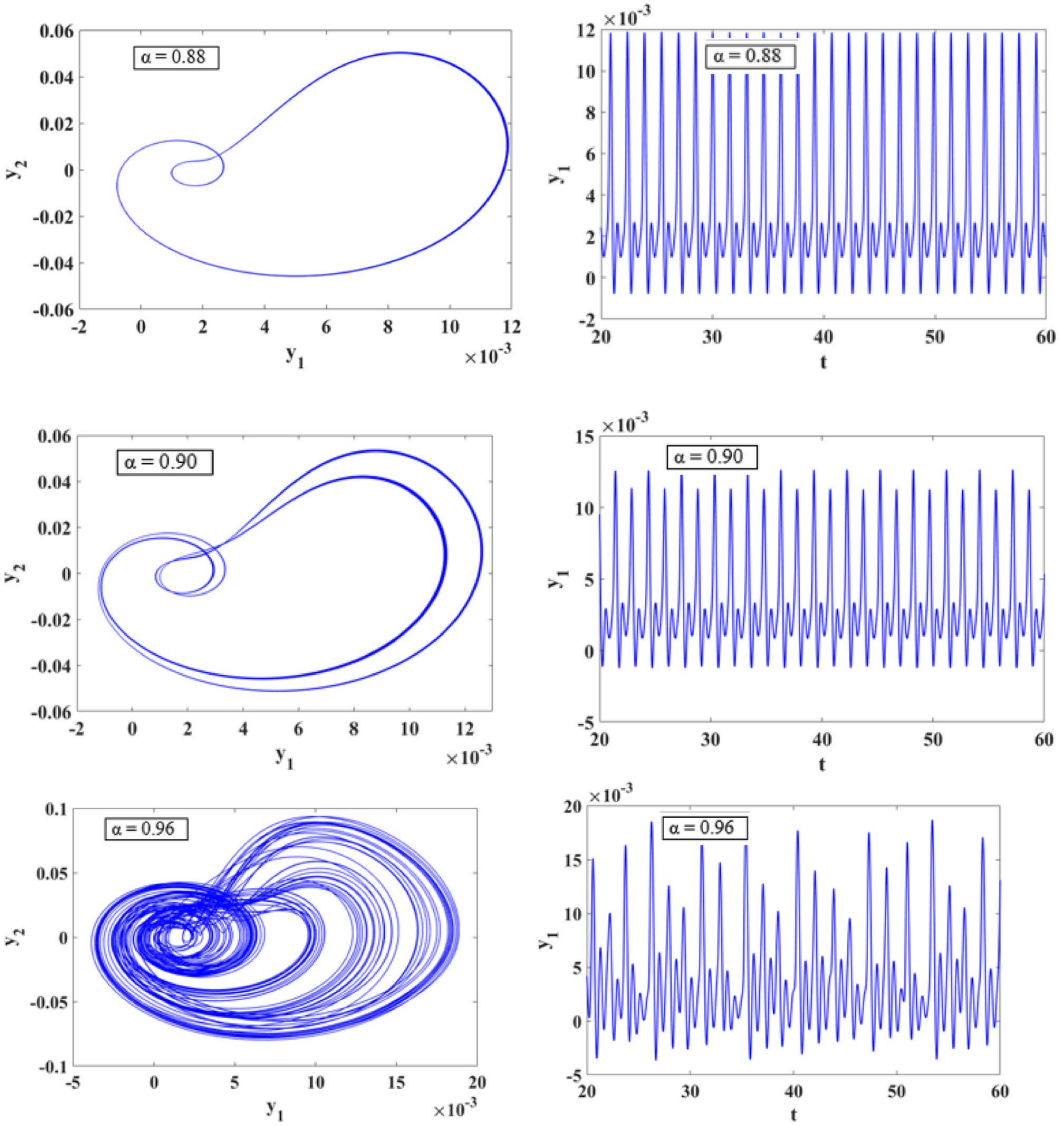
The dynamic response of the state variables of system (12) for various values of $\alpha = 0.88,\,0.90,\,0.96.$

**Figure 13:**
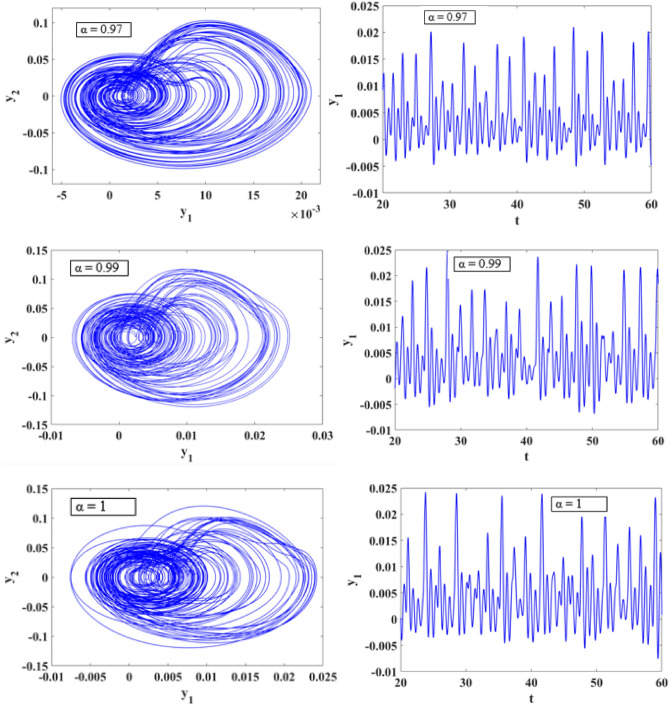
The dynamic response of the state variables of system (12) for various values of $\alpha = 0.97,\,0.99,\,1.$

In this section we analyze the dynamical behavior of the suspension system and presented the numerical simulations of fractional order system (12) in the presence of stochastic excitation. For the initial conditions [0, 0.1, 0. 1, 0.1, 0.1] and fractional order $\alpha = 0.88,\,0.90,\,0.96,\,0.97,\,0.99,\,1$ the 2D state portraits of the system are given in Figs. 12 and 13. We can observe that for α = 0.88, a clear periodic oscillation and while increasing the $\alpha$ value model exhibits chaotic oscillations. These findings confirm that fractional-order models provide a more accurate representation of nonlinear systems with complex dynamics compared to their integer-order counterparts.

### Bifurcation plot and Lyapunov spectrum

To gain insight into the dynamic behavior of the nonlinear quarter car model, we investigate bifurcation diagrams in the two cases of commensurate-order and incommensurate-order, respectively. Bifurcation diagrams are a commonly used technique for visually depicting how dynamic patterns change across a spectrum of parameter values. They are particularly useful for illustrating the transitions from periodic to chaotic motion in dynamic systems.

#### Case (A) for an incommensurate system (12)

**Figure 14:**
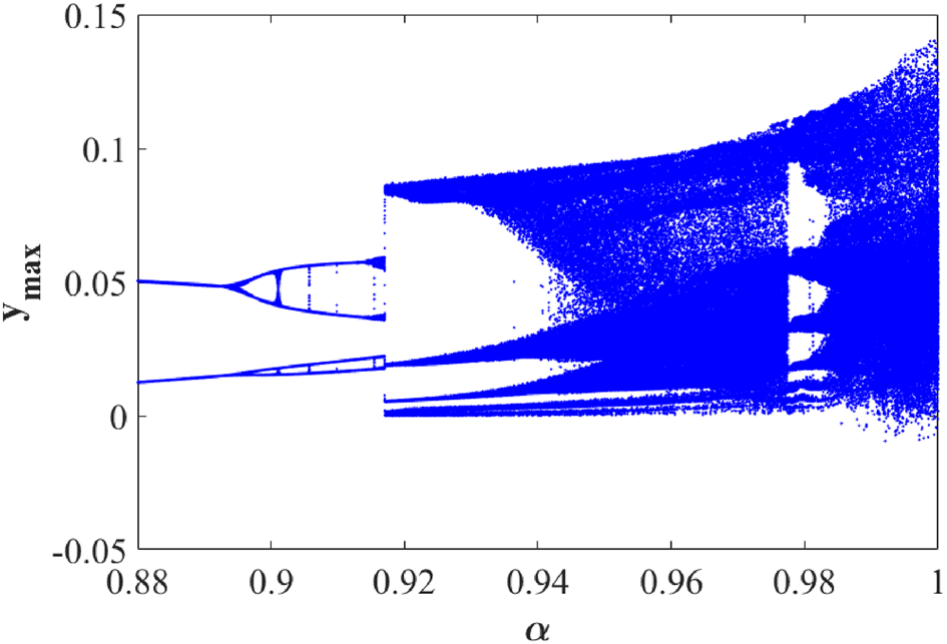
Bifurcation diagram of the system (12) for changes of parameter α.

Compared with an integer-order system, the derivative order is an important parameter for a fractional-order system. For system (12), the system parameters and the initial conditions are fixed. The bifurcation diagrams with different values of the derivative order $\alpha \in \left( {0.88,1} \right)$ and a time step of $h = 0.001$ are employed to demonstrate the behavior of system (12), as shown in Fig. 14. From which it is evident that within the range of 0.895 ≤ α ≤ 0.9169, the system exhibits period doubling, while for α values in the range of 0.917 ≤ α ≤ 1.000, the suspension system displays chaotic vibrations.

#### Case (B) for a commensurate system (12)

**Figure 15:**
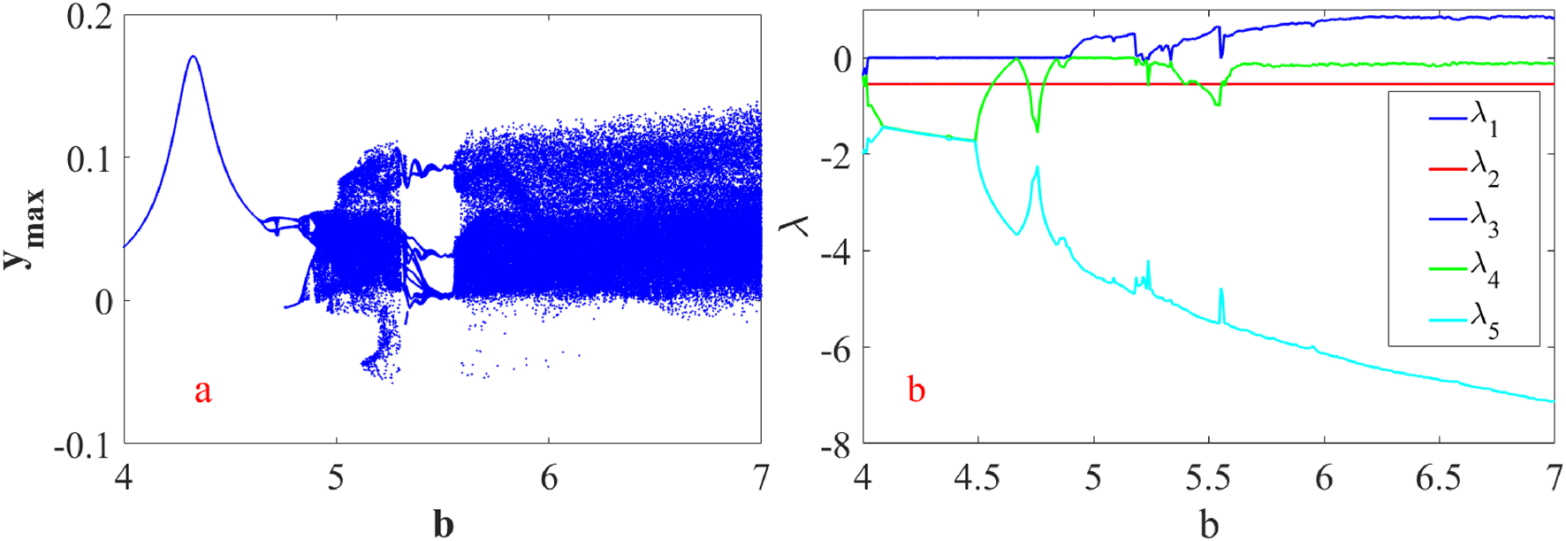
The bifurcation and spectrum for the system (12) (**a**) bifurcation and (**b**) Lyapunov spectrum for α = 0.998.

Bifurcations with the variation of the system parameter *b* ∈ (4,7) are studied for α = 0.998, bifurcation diagram for system (12) is depicted in Fig. 15a. Clearly, the evolution of chaotic solutions and the period-doubling scenario bifurcation can be observed from this figure. The system indicates a period doubling bifurcation at *b* = 4.653 and two regions of chaotic oscillations for 4.83 ≤ *b* ≤ 5.3, 5.549 ≤ *b* ≤ 7. The corresponding Lyapunov spectrum as shown in Fig. 15b.

### Discussion

This research delves into examining the dynamic behavior of single degree of freedom (SDOF) nonlinear quarter-car models by incorporating fractional order dynamics. By selecting a fractional order parameter $\alpha$ of 0.998, the study ensures that the analysis of phase portraits, Lyapunov exponents, eigenvalues, and parameter-dependent bifurcations remains within the framework of fractional calculus. Figures 12 and 13 visually depict the evolution of phase trajectories, revealing their intricate nature, while also demonstrating non-periodic behavior in the time-domain response. The emergence of non-periodicity is particularly noteworthy when the largest Lyapunov exponent, represented as $L_{3} = 0.7675$, takes on a positive value. This observation underscores the ability of fractional order dynamics to unveil chaotic regions at earlier stages compared to systems governed by integer order dynamics, especially as α approaches 1.

Furthermore, Fig. 12 provides a comprehensive illustration of the system’s response across varying α values. Within the α range of 0.88 to 0.9, the system undergoes period doubling, a phenomenon indicating a transition from periodic to quasi-periodic motion. Conversely, when α falls within the range of 0.97 to 1.000, the suspension system exhibits chaotic vibrations, representing a complex and unpredictable behavior as shown in Fig. 13. The bifurcation diagram presented in Fig. 15a clearly illustrates a period doubling bifurcation occurring at $b = 4.653$, along with two regions characterized by chaotic oscillations within the parameter ranges of $4.83 \le b \le 5.3$ and $5.549 \le b \le 7$.

Moreover, the correlation between the observed behaviors and the Lyapunov spectrum, as depicted in Fig. 15b, reinforces the findings obtained from the analysis. The Lyapunov spectrum offers valuable insights into the stability properties of the system, with distinct regions corresponding to different dynamic behaviors. By examining the Lyapunov spectrum alongside the phase portraits and bifurcation diagrams, a holistic understanding of the system’s behavior across various fractional order parameters emerges, facilitating a deeper exploration of its dynamical characteristics and offering valuable implications for practical applications and system design.

## Conclusion

We investigated the complex dynamics found in automotive suspension systems in this study, with a particular emphasis on quarter car models. The experimental approach illustrated the presence of nonlinearity in quarter car models and its substantial impact on the real suspension system. This result highlights how crucial it is to account for nonlinear effects in suspension system modelling and analysis in order to ensure precise representation and optimization of a nonlinear quarter car dynamics. The study investigated the dynamic characteristics of the proposed fractional-order nonlinear quarter car model, utilizing Caputo derivatives, and employed the Adam–Bashforth–Moulton (ABM) numerical method to investigate the system behaviour. Furthermore, the exploration of fractional order calculus has introduced a more nuanced mathematical framework capable of capturing the intricate memory effects and complex interactions inherent in suspension systems. This research underscores the practical relevance of considering real-world road conditions in suspension system studies, providing a robust foundation for future improvements aimed at enhancing overall performance. The research underscores the critical importance of embracing nonlinearity and fractional order calculus in advancing the field of vehicular suspension systems.

## Future work

Further research directions are suggested, building on the knowledge obtained from this investigation. The focus of future research will be on carefully studying the intricate interaction present in two parameter bifurcation in the hope of gaining deep understanding of the dynamics of complex systems. Future studies should delve deeper into dynamical analysis of two degrees of freedom vehicle systems. Future research will focus on the application of real-time control design based on fractional order dynamics representation for the suppression of chaos.

## Data Availability

The data used to support the findings of this paper are included within the manuscript.
